# Validation of Neutrophil Count as An Algorithm-Based Predictive Factor of Progression-Free Survival in Patients with Metastatic Soft Tissue Sarcomas Treated with Trabectedin

**DOI:** 10.3390/cancers11030432

**Published:** 2019-03-26

**Authors:** Alexandre de Nonneville, Dominique Barbolosi, Maeva Andriantsoa, Raouf El-Cheikh, Florence Duffaud, François Bertucci, Sebastien Salas

**Affiliations:** 1 CRCM, Department of Medical Oncology, Institut Paoli-Calmettes, INSERM, CNRS, Aix-Marseille University, 13009 Marseille, France; bertuccif@ipc.unicancer.fr; 2 SMARTc Unit, CRCM Inserm U1068, Aix-Marseille Université, 13005 Marseille, France; dominique.barbolosi@univ-amu.fr (D.B.); elcheikhraouf@gmail.com (R.E.-C.); 3 Department of Medical Oncology, Hôpital de la Timone, APHM, INSERM U910, Aix-Marseille University, 13005 Marseille, France; maeva.andriantsoa@gmail.com (M.A.); Florence.DUFFAUD@ap-hm.fr (F.D.); Sebastien.SALAS@ap-hm.fr (S.S.)

**Keywords:** mathematical modelling, neutrophil, trabectedin, soft tissue sarcomas, predictive marker

## Abstract

*Introduction*: Based on a mathematical model of trabectedin-induced neutropenia, we assessed the predictive value of absolute neutrophil count (ANC) on progression-free survival (PFS) in an independent validation cohort of patients treated with trabectedin. *Methods*: We collected data from 87 patients in two expert centers who received at least two cycles of trabectedin for soft tissue sarcomas (STS) treatment. Correlations between ANC, patients’ characteristics, and survival were assessed, and a multivariate model including tumor grade, performance status, ANC, and hemoglobin level was developed. *Results*: Therapeutic ANC ≥ 7.5 G/L level was associated with shorter PFS: 3.22 months (95% confidence interval (CI), 1.57–4.87) in patients with ANC ≥ 7.5 G/L vs. 5.78 months (95% CI, 3.95–7.61) in patients with ANC < 7.5 G/L (*p* = 0.009). Age, primary localization, lung metastases, dose reduction, hemoglobin, and albumin rates were also associated with PFS. In multivariate analysis, ANC ≥ 7.5 G/L was independently associated with poor PFS and overall survival. Conclusion: We validated increased pre-therapeutic ANC as a predictive factor of short PFS in patients starting trabectedin for STS. ANC appears to have an impact on survival rates and may be used as a decision-making tool for personalizing second-line strategies in patients with metastatic STS.

## 1. Introduction

Soft tissue sarcomas (STS) are rare malignancies of mesenchymal origin, accounting for approximately 1% of adult cancers [1]. In patients with metastatic disease not amenable to curative-intent surgery, the first-line systemic treatment involves palliative doxorubicin-based chemotherapy, which has changed very little over the past three decades. After intolerance or failure, the second-line therapies include chemotherapies (trabectedin, ifosfamide, dacarbazine) and targeted therapies (pazopanib, olaparumab), but the results remain disappointing. Clearly, the improvement of systemic therapies is crucial, including the identification of predictive factors for response to specific drugs.

Trabectedin is a synthetic antineoplastic agent, derived from a sea squirt. It has demonstrated efficacy in several randomized phase II studies [2,3,4] in non-selected patients with advanced, unresectable disease and no previous exposure to chemotherapy [5], who are in compassionate programs [6,7,8]. Furthermore, a very recent, randomized phase III study showed a significant improvement in median progression-free survival (PFS) with trabectedin vs. Best Supportive Care, in patients with pre-treated, advanced STS of multiple histological types [9]. Liposarcoma-STS patients benefit the most from trabectedin therapy in terms of prolonged tumor control. Identification of the predictors of response to trabectedin is warranted, as well, because of its morbid and financial toxicity. 

One of the main toxicities of trabectedin is neutropenia. The incidence of grade 3 and 4 neutropenia has been estimated at 26% and 24%, respectively. Although reversible, trabectedin-induced neutropenia represents a major concern for treatment continuation and optimal dose delivery. In order to develop an alternative administration schedule, in which similar doses would be less hematotoxic, we recently developed an original, mathematical pharmacokinetics/pharmacodynamics (PK/PD) model describing the time course of neutrophils, under trabectedin treatment, in a monocenter retrospective series of 39 patients with metastatic STS [10]. Model parameters were estimated using a nonlinear mixed effects analysis with MONOLIX software (Lixoft, Anrony, France). A visual inspection of the baseline absolute neutrophil count (ANC) revealed the existence of two subpopulations of patients: one with a baseline ANC ≥ 7.5 G/L and one with ANC < 7.5 G/L. To assess the validity of this hypothesis, a latent covariate structure was introduced at the individual parameter level, namely on ANC at baseline. This was accomplished using the between-subject mixture option in MONOLIX. Population analysis results confirmed the existence (*p* < 0.005 for the latent covariate) of two distinct distributions for the parameter representing ANC at baseline, thus confirming the existence of two subpopulations of patients, distinguishable by their baseline ANC. Importantly, progression-free survival (PFS) was significantly lower in the group with ANC ≥ 7.5 G/L (hazard ratio (HR) = 3.819; 95% confidence interval (CI) (0.44–32.96); *p* = 0.015).

The primary objective of the present study was to validate the impact of pre-therapeutic ANC on PFS in an independent external cohort of STS patients treated with trabectedin. Secondary objectives consisted of an exploratory analysis of the impact of pre-therapeutic ANC on overall survival (OS) and the identification of other predictive factors for PFS and OS. 

## 2. Results

### 2.1. Characteristics of Study Population

A total of 87 patients who received trabectedin for metastatic STS were considered eligible for analysis. Baseline characteristics are reported in Table 1. The most prevalent histologic type was leiomyosarcomas, followed by liposarcomas, and undifferentiated sarcomas, with 17% of total cases corresponding to translocation-related sarcomas (myxoid liposarcoma: 40%, synovialosarcomas: 34%, rhabdomyosarcoma: 13%, clear cell sarcoma: 7%, and Ewing sarcoma: 6%). Primary tumors were located in a limb in 33% of cases. Other localizations were variable, including visceral localizations (uterine: 19%; digestive: 7%) and primitive serous sarcomas (5%). Lung was the most common site of metastatic disease, followed by liver and bone.

A median of five cycles of trabectedin were administered (range 2–73). Dose reduction occurred in 73% of patients either during the first cycle, due to initial fragility, or during treatment, due to trabectedin-related toxicities. Pre-therapeutic assessment found an initial ANC < 7.5 in 59 patients (80%) and ≥7.5 in 15 patients (20%). Baseline characteristics did not differ according to these two ANC-based subgroups (Table 1).

### 2.2. Impact of Pre-Therapeutic ANC on PFS

**Figure 1:**
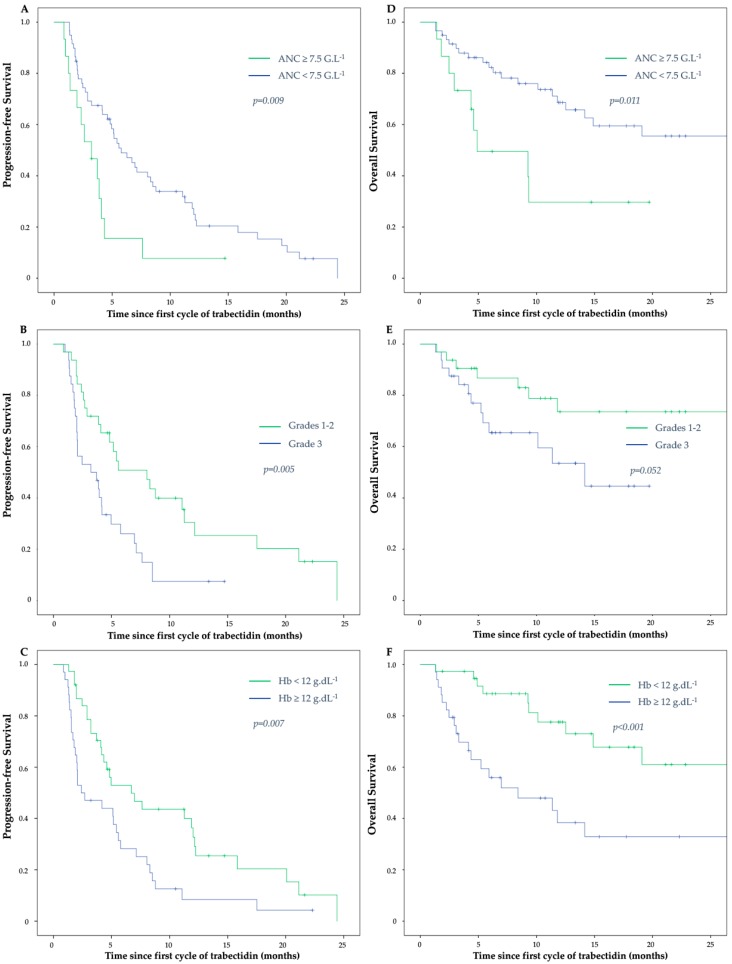
Kaplan-Meier estimates of progression-free survival (left panels) and overall survival (right panels), respectively, in patients with absolute neutrophil count (ANC) < or ≥ 7.5 G/L (**A**,**D**) patients with grade 1, 2, or 3 tumors (**B**,**E**) and patients with hemoglobin level (Hb) < or ≥ 12 g/L (**C**,**F**).

**Figure 2:**
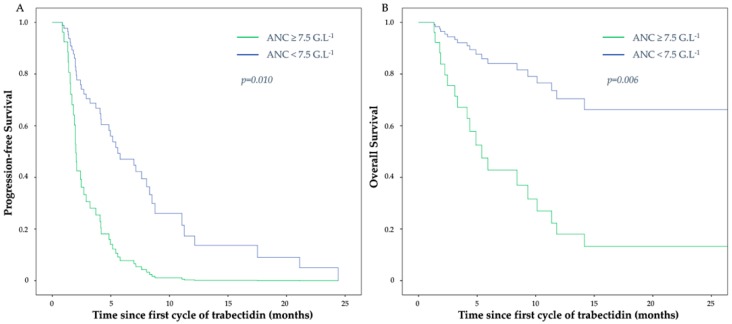
Cox-adjusted curves of progression-free survival (**A**) and overall survival (**B**) in patients with ANC < or ≥ 7.5 G/L.

After a median follow-up of 9.26 months (range 1.31–52.86), 74 patients (85%) had progressive disease. Median PFS of the overall population was 4.17 months (95% CI, 3.10–5.24). In univariate analysis, a pre-therapeutic ANC ≥ 7.5 G/L was associated with a shorter median PFS of 3.22 months (95% CI, 1.57–4.87) vs. 5.78 months (95% CI, 3.95–7.61) in patients with ANC < 7.5 G/L (*p* = 0.009) (Figure 1A). Age, tumor grade (Figure 1B), primary tumor localization, lung metastases, trabectedin dose reduction, hemoglobin level (Figure 1C), and albumin level also impacted PFS (Table 2). The predictive negative effect of ANC ≥ 7.5 G/L on PFS was independently maintained in multivariate analysis (HR = 3.39) (95% CI, 1.35–8.53; *p* = 0.010) (Figure 2A), as well as high tumor grade and low hemoglobin level (Table 3).

### 2.3. Impact of Pre-Therapeutic ANC on Overall Survival: An Exploratory Analysis

Thirty-nine deaths (45%) occurred during the follow-up. The median OS of the overall population was 26.78 months (95% CI, 14.38–39.17). In univariate analysis, a pre-therapeutic ANC ≥ 7.5 G/L was associated with a shorter median OS of 4.89 months (95% CI, 0–11.81) vs. 26.78 months (95% CI, 13.47–40.08) in patients with ANC < 7.5 G/L (*p* = 0.011) (Figure 1D). Eastern Cooperative Oncology Group (ECOG) performance status, hemoglobin level (Figure 1F), and albumin level also impacted OS (Table 2). Despite a clear trend, tumor grade did not reach statistical significance (Figure 1E). The predictive negative effect of ANC ≥ 7.5 G/L on OS was independently maintained in multivariate analysis (HR = 4.90 (95% CI, 1.57–15.34); *p* = 0.006) (Figure 2B), as well aslow hemoglobin level (Table 3). As in univariate analysis, tumor grade failed to reach statistical significance (HR = 2.49 for grade 3 (95% CI, 0.92–6.73); *p* = 0.071).

## 3. Discussion

In this assessment of the predictive value of pre-therapeutic ANC on PFS, in an independent validation cohort of patients treated with trabectedin for metastatic STS, an ANC ≥ 7.5 G/L was strongly associated with poorer PFS. Our previous mathematical model of trabectedin-induced neutropenia had shown that pre-therapeutic ANC could discriminate two populations of patients with different survival rates. It revealed a predictive factor that we would not necessarily have intuitively identified, and allowed us to choose the right threshold for validation as a future decision-making parameter. Using mathematical models to improve cancer treatment is a strategy on the rise, encompassing model-driven dosing and scheduling, and leading to the development of algorithms for precision medicine [11]. Median PFS of the overall population in our cohort was very similar to the pivotal, multicenter phase III trial comparing trabectedin and dacarbazine (4.2 months in the trabectedin arm) [12]. In the T-SAR trial, the median PFS was 1.5 months (m) in the best supportive care (BSC) arm and 3.1 m in the trabectedin arm. In the liposarcoma-STS cohort, the median PFS was 1.4 m and 5.1 m in the BSC and trabectedin arms, respectively, whereas it was 1.5 m and 1.8 m in the non-liposarcoma-STS group. In our study, histologic type had no prognostic value, although liposarcomas represented only 18% of all cases. 

Although high histological grade is widely recognized as a factor of poor prognosis [13,14,15,16,17], high pre-therapeutic ANC outperformed its prognostic value in multivariate analysis. The independent negative prognostic value of high ANC has already been described in other cancer types, regardless of trabectedin use, notably in non-small-cell lung carcinomas [18], squamous oropharyngeal carcinomas [19], and melanoma [20], with a range of optimal thresholds from 4.5 to 7.5 G/L. Recent data showed that trabectedin selectively targets tumor-associated macrophages (TAM), and decreases the production of pro-inflammatory mediators, thus modifying the tumor microenvironment. This mode of action may contribute to the antitumor and anti-angiogenic activities of trabectedin [21,22,23,24]. Although neutrophilia is often present at cancer diagnosis, the biological pathways that may explain this observation have not been clearly identified and the origin appears to be multifactorial. One of these could be the paraneoplastic production of hematopoietic growth factors by tumor cells. Several studies have shown the existence of autocrine loops in the production of granulocyte growth factors (G-CSF and GM-CSF) in these tumors [25,26,27,28,29]. Interleukin-6 (IL-6) and tumor necrosis factor-alpha (TNFα), which are involved in tumor-related inflammatory processes, are known to induce neutrophilia independently [30,31,32]. Tumor inflammation could lead to an antitumor immunity deficiency by involving T-helpers and T-regulating lymphocytes, notably via immune checkpoints (PD1–PDL1, CTLA4) and cytokines [33,34], thus promoting tumor growth. Another hypothesis is that neutrophils directly inhibit cellular immunity and, therefore, have an impact on the host antitumor response. In vitro studies have shown that an increased ANC inhibits the cytolytic activity of lymphocytes and (natural killer) NK cells [35,36,37]. Our study validated ANC ≥ 7.5 G/L as a negative predictive factor for PFS (HR = 3.39 (95% CI, 1.35–8.53); *p* = 0.010).

High histological grade and Hb < 12 g/dL were also independently associated with poor PFS. Although high histological grade is already recognized as a negative prognostic factor, we found that this initial prognostic value is still maintained in heavily pre-treated metastatic STS. Other prognostic factors of STS have already been identified, including in the early stages, such as tumor size and depth, primary and metastasis sites, patient’s age at diagnosis, gender, ECOG performance status, and previous treatments for advanced STS level [38,39,40,41,42,43,44,45,46,47,48]. Hemoglobin level has a prognostic value in metastatic renal cell carcinoma, as part of the International Metastatic Renal Cell Carcinoma Database Consortium (IMDC) score [49], and tends to be associated with shorter OS in lung cancer patients [50]. Nevertheless, there is no data on the prognostic value of baseline Hb levels and outcomes in sarcomas. The increasingly widespread use of molecular biology is now promoting a better understanding of the complexity of STS and making it possible to identify predictive signatures of the risk of metastatic recurrence in localized STS [51,52]. In vitro studies have suggested that cells with a deficient homologous recombination DNA repair pathway have a higher sensitivity to trabectedin [53], and retrospective studies have shown that BRCA1 (breast cancer gene 1) status may be predictive of trabectedin efficacy [54,55,56]. This observation was very recently assessed in a prospective EORTC (European Organisation for Research and Treatment of Cancer) study comparing the efficacy of trabectedin to doxorubicin, as a first line for advanced/metastatic soft tissue sarcoma [57]. It failed to confirm the predictive value of BRCA1 haplotype for trabectedin efficacy, but the number of informative patients was limited and there was no assessment of BRCA1 expression levels.

The six-month, progression-free rate with trabectedin is about 35–40% [12]. Thus, it is essential to establish predictive biomarkers of the clinical benefit of trabectedin to identify potential responders. This would offer a long-term tumor stabilization strategy for such patients and avoid treating those who would, potentially, only have side-effects with it. In our study, the PFS of patients with high ANC was equivalent to the PFS of liposarcoma patients in the trabectedin arm of the T-SAR trial, yet our cohort included only a minor proportion of liposarcomas. Baseline ANC may help in selecting candidates who would benefit from trabectedin treatment for advanced STS, leaving a gemcitabine-docetaxel regimen for those with higher ANC levels. Pazopanib, eribulin, aldoxorubicin, and regorafenib are other therapeutic options for use in advanced STS [58]. In our exploratory analysis of OS, we demonstrated here that high ANC could even be predictive of poorer OS. As discussed, high ANC could reflect tumor-related inflammatory processes involving T-helpers and T-regulating lymphocytes, or those directly inhibiting cellular immunity such as the paraneoplastic production of hematopoietic growth factors. This might reflect individual variations with regard to disease progression and patients’ general condition.

Our study is limited by its retrospective design due to a potential selection bias and lack of standardization in previous treatment strategies. However, the results concern a multicenter cohort of real-life patients treated in two expert centers and are, therefore, more likely to reflect the true diversity of patients treated by trabectedin in routine clinical practice. 

## 4. Materials and Methods

### 4.1. Study Design and Data Source

We conducted a retrospective analysis of patients treated with trabectedin between January, 2010 and February, 2018 in two French, academic cancer centers. Because of the observational, non-interventional nature of this work, and since all patients were treated according to current guidelines such that their care was not affected or modified by the study, no ethical committee approval or clinical trial number was necessary under French law. All patients had metastatic STS and received at least two cycles of trabectedin, at the recommended dose of 1.5 mg/m² in 24-hour infusion every 21 days, as a standard therapy or in a clinical trial. Patient and tumor characteristics (i.e., age, sex, ECOG performance status (PS), body mass index (BMI), histological type, FNCLCC tumor grade (Fédération Nationale des Centres de Lutte Contre le Cancer), primary tumor location, existence of pulmonary, bone and/or hepatic metastases, pre-trabectedin ANC, hemoglobin and albumin levels, and progression and death, if any), were retrieved from medical files. Systemic treatment data (number of cycles, initial dose of trabectedin and eventual dose reduction, and previous and/or following treatment line, if any) were extracted from common computerized prescription software in our institutions (Chimio^®^, Computer Engineering, Paris, France).

### 4.2. Statistical Analysis

Categorical variables were described using counts and frequencies, and quantitative variables were reported using median and range. Patient characteristics were compared with the χ^2^ test for categorical variables and with the rank-Wilcoxon test for quantitative variables. Some variables were categorized according to cut-off values: age ≥ or <65 years, PS ECOG ≤ or >1, BMI < 19, between 19 and 25 or >25, number of prior chemotherapy lines > or ≤2, PFS under previous chemotherapy regimen, if any > or ≤4 months, ANC ≥ or <7.5 G/L, Hb level ≥ or <12 g/dL, and albumin level ≥ or <35 g/L, but also histological type (leiomyosarcoma, liposarcoma, undifferentiated sarcoma, and others), FNCLCC grade (1–2 or 3), and location of primary tumor (limb or other). PFS was defined as the time from the first course of trabectedin, to clinical and/or radiological progression, or to death from any cause, dependent on whichever occurred first. OS was defined as the time from the first course of trabectedin to death from any cause. Survival curves for PFS and OS rates were generated using the Kaplan-Meier method and compared with log-rank tests. The independent prognostic impact of the different clinico-biological factors identified in univariate analysis (*p* < 0.05) was tested by a multivariate Cox regression model including the following covariates categorized as mentioned above: performance status, tumor grade, ANC, and hemoglobin level.

The level of statistical significance was set at α = 0.05. Statistical analyses were carried out with SPSS^®^ software version 24 (IBM, Armonk, New York, USA) and R software version 3.2.4. (R Foundation for Statistical Computing, Vienna, Austria). We followed the reporting recommendations specified in the REMARK (Reporting recommendations for tumor Marker prognostic studies) Statement [59].

## 5. Conclusions

We have validated high (i.e., >7.5) pre-therapeutic ANC as a predictive factor of poor PFS in patients starting trabectedin for STS. ANC could thus serve as a simple and inexpensive pre-therapeutic marker for decision-making in patients with metastatic STS.

## Figures and Tables

**Table 1:** Patient and tumor characteristics. Percentages are calculated in relation to the number of available data.

Variables	Total Population		Patients According to Pre-Therapeutic ANC				
			ANC < 7.5 G/L		ANC ≥ 7.5 G/L		*p*-Value
	*n*	%	*n*	%	*n*	%	(Khi2 or Wilcoxon)
Gender							
Female	49	56%	37	63%	6	40%	0.111
Male	38	44%	22	37%	9	60%	
Age (years)							
Median (Min–Max)	59.75	(19.96–83.58)	59.75	(22.03–83.58)	55.32	(19.96–70.13)	0.480
<65 y	62	71%	42	71%	10	67%	0.732
≥65 y	25	29%	17	29%	5	33%	
ECOG status							
<2	68	81%	11	19%	4	29%	0.446
≥2	16	19%	46	81%	10	71%	
MD	3						
Histological type							
LMS	38	44%	26	44%	4	27%	0.672
LPS	16	18%	11	19%	4	27%	
UPS	4	5%	3	5%	1	7%	
OTHER	29	33%	19	32%	6	40%	
Translocation-related sarcoma							
No	72	83%	49	83%	11	73%	0.391
Yes	15	17%	10	17%	4	27%	
Grade (FNCLCC)							
G1	4	6%	2	4%	2	18%	0.164
G2	28	44%	23	51%	3	27%	
G3	32	50%	20	44%	6	55%	
MD	23						
Location of the primary site							
Limb	29	33%	20	34%	5	33%	0.967
Other	58	67%	39	66%	10	67%	
Lung metastases							
No	23	27%	16	28%	3	20%	0.551
Yes	63	73%	42	72%	12	80%	
MD	1						
Liver metastases							
No	54	63%	36	62%	12	80%	0.192
Yes	32	37%	22	38%	3	20%	
MD	1						
Bone metastases							
No	63	75%	41	73%	11	73%	0.993
Yes	21	25%	15	27%	4	27%	
MD	3						
BMI							
<19	14	16%	9	16%	3	20%	0.914
19–25	41	48%	28	48%	7	47%	
>25	31	36%	21	36%	5	33%	
MD	1						
Number of prior lines of chemotherapy							
Median (Min–Max)	2	(0–7)	2	(0–5)	3	(0–7)	0.269
≤2	54	64%	39	67%	6	43%	0.091
>2	31	36%	19	33%	8	57%	
MD	2						
PFS under previous chemotherapy regimen							
Median (Min–Max) (months)	5.09	(0.29–20.53)	5.06	(1.61–19.81)	3.43	(0.76–20.53)	0.103
≤4 months	33	48%	23	49%	7	58%	0.561
>4 months	36	52%	24	51%	5	42%	
MD	18						
Trabectedin dose reduction							
No	23	27%	16	27%	4	29%	0.913
Yes	63	73%	43	73%	10	71%	
MD	1						
Absolute neutrophil count at the 1st cycle of Trabectedin							
<7.5	59	80%					
≥7.5	15	20%					
MD	13						
Hemoglobin at the 1st cycle of Trabectedin							
<12	34	47%	26	46%	5	42%	0.803
≥12	38	53%	31	54%	7	58%	
MD	15						
Albumin at the 1st cycle of Trabectedin							
<35	17	41%	9	32%	6	60%	0.122
≥35	24	59%	19	68%	4	40%	
MD	46						

Abbreviation: LMS, Leiomyosarcoma; LPS, Liposarcoma; UPS, Undifferentiated pleomorphic sarcoma; BMI, Body Mass Index; PFS, Progression-free survival; MD, Missing data.

**Table 2:** Univariate analysis of factors associated with progression-free survival (PFS) and overall survival (OS).

Variables	Classes	Median PFS (CI 95%)	*p*-Value Log-Rank	Median OS (CI 95%)	*p*-Value Log-Rank
Sex	Male	4.07 (2.51–5.65)	0.355	17.18 (5.08–29.28)	0.787
	Female	4.83 (3.44–6.22)		26.78 (12.70–40.85)	
Age	≥65	5.78 (0.15–11.41)	0.038	27.50 (27.10–27.90)	0.135
	<65	4.07 (2.56–5.58)		14.88 (8.21–21.55)	
Performance Status	≤1	4.17 (2.27–6.07)	0.99	27.30 (13.63–40.97)	0.015
	>1	3.94 (2.41–5.48)		6.96 (0.88–13.05)	
Histological type	LMS	5.42 (2.89–7.95)	0.15	27.49 (25.39–29.60)	0.126
	LPS	4.07 (0.49–7.66)		6.97 (3.65–10.28)	
	UPS	1.54 (0.84–2.25)		5.91 (0.405–11.42)	
	OTHER	3.88 (2.32–5.43)		17.18 (10.06–24.31)	
Translocation-related sarcoma	Yes	3.71 (1.46–5.97)	0.855	14.88 (9.76–29.73)	0.959
	No	4.60 (3.31–5.89)		26.78 (14.93–38.63)	
Grade (FNCLCC)	G1	4.07 (0–8.29)	0.008	4.89 (0–21.94)	0.052
	G2	8.31 (2.15–14.47)		28.78 (26.28–31.28)	
	G3	3.22 (0.76–5.68)		14.160 (7.77–20.55)	
Location of the primary site	Limb	5.78 (2.11–9.45)	0.028	33.12 (9.05–57.18)	0.184
	Other	3.94 (2.45–5.43)		17.18 (9.8–24.56)	
Lung metastases	Yes	3.88 (2.55–5.19)	0.029	26.78 (5.78–47.77)	0.090
	No	11.89 (2.43–21.35)		45.68 (8.54–59.42)	
Liver metastases	Yes	4.60 (2.60–6.60)	0.667	28.78 (19.92–37.64)	0.145
	No	3.88 (2.25–5.51)		14.88 (6.38–23.39)	
Bone metastases	Yes	3.22 (1.16–5.28)	0.292	14.88 (3.60–26.16)	0.297
	No	4.34 (3.07–5.60)		26.78 (15.44–38.10)	
BMI	<19	2.04 (1.43–2.64)	0.589	11.37 (3.08–19.66)	0.388
	19–25	4.17 (2.85–5.49)		27.30 (11.05–43.56)	
	>25	5.16 (3.44–6.87)		26.78 (4.69–48.86)	
Number of prior lines of chemotherapy	>2	3.71 (2.00–5.42)	0.254	17.18 (3.38–30.99)	0.814
	≤2	4.83 (3.07–6.59)		27.30 (13.95–40.65)	
Trabectedin dose reduction	Yes	5.42 (3.48–7.36)	0.018	27.30 (16.25–38.35)	0.484
	No	2.89 (1.91–3.87)		14.88 (8.59–21.18)	
Absolute neutrophil count at the 1st cycle of Trabectedin	>7.5	3.22 (1.60–4.87)	0.009	4.89 (0–11.82)	0.011
	≤7.5	5.78 (3.95–7.61)		26.78 (13.47–40.08)	
Hemoglobin at the 1st cycle of Trabectedin	≥12	6.70 (2.99–10.42)	0.007	43.14 (17.87–68.40)	<0.001
	<12	1.40 (0–4.81)		8.41 (0.66–16.16)	
Albumin at the 1st cycle of Trabectedin	≥35	4.83 (3.89–5.77)	0.043	26.78 (10.06–43.49)	0.036
	<35	2.07 (1.37–2.77)		4.89 (1.39–8.40)	

**Table 3:** Multivariate analysis of factors associated with progression-free survival (PFS) and overall survival (OS).

Variables	PFS				OS			
	HR	(95% CI)		*p*-Value	HR	(95% CI)		*p*-Value
		min	max			min	max	
Performance Status								
1–2	Reference category							
3–4	1.52	0.70	3.30	0.289	1.00	0.37	2.74	0.998
Grade (FNCLCC)								
1–2	Reference category							
3	2.75	1.40	5.40	0.003	2.49	0.92	6.73	0.071
Absolute neutrophil count								
<7.5 G/L	Reference category							
≥7.5 G/L	3.39	1.35	8.53	0.010	4.90	1.57	15.34	0.006
Haemoglobin								
<12 g/dL	Reference category							
≥12 g/dL	0.35	0.17	0.72	0.005	0.17	0.06	0.50	0.001

FNCLCC: Fédération Nationale des Centres de Lutte Contre le Cancer; HR: hazard ratio.
